# Utility of a Computerized Cognitive Function Assessment Tool (CogEvo) in Patients With Acute Stroke: Associations With the Mini-Mental State Examination, Trail Making Test, and Length of Hospital Stay

**DOI:** 10.7759/cureus.111504

**Published:** 2026-06-25

**Authors:** Keiichiro Aoki, Takeaki Miyata, Akihiro Iguchi, Miho Akimoto, Aya Osawa

**Affiliations:** 1 Department of Occupational Therapy, School of Rehabilitation, Tokyo Professional University of Health Sciences, Tokyo, JPN; 2 Department of Rehabilitation, Showa Medical University, Yokohama, JPN

**Keywords:** acute stroke, cogevo, cognitive screening, ict assessment, length of hospital stay, mmse, occupational therapy, trail making test

## Abstract

Background: Cognitive impairment is a common and clinically significant consequence of acute stroke, frequently associated with prolonged hospitalization and poor functional outcomes. Conventional paper-based tools such as the Mini-Mental State Examination (MMSE) and Trail Making Test (TMT) have recognized limitations in the acute setting, including ceiling effects and high rates of non-completion. CogEvo, a tablet-based computerized cognitive assessment tool evaluating five domains (orientation, attention, memory, planning, and spatial cognition), offers a potentially feasible alternative; however, its applicability in acute stroke has not been established.

Methods: In this prospective single-center pilot study, 17 consecutive acute stroke patients underwent concurrent CogEvo, MMSE, and TMT-A/B assessments during occupational therapy evaluation (mean 13.9 ± 7.5 days post-admission). Spearman rank-order correlations were computed between CogEvo composite and domain scores and MMSE, length of hospital stay, TMT-A/B, National Institutes of Health Stroke Scale (NIHSS), and age. TMT analyses used separate complete case analyses (TMT-A: N = 12; TMT-B: N = 10).

Results: All 17 patients completed CogEvo; only 10 (58.8%) completed TMT-B. CogEvo composite scores were significantly correlated with MMSE (r = 0.693, p = 0.002), length of hospital stay (r = −0.518, p = 0.033), TMT-A (r = −0.580, p = 0.048, N = 12), and TMT-B (r = −0.648, p = 0.043). Domain-level analyses (N = 14) revealed that attention (r = 0.633, p = 0.015), planning (r = 0.668, p = 0.009), and spatial cognition (r = 0.664, p = 0.010) were significantly correlated with MMSE; planning was also significantly correlated with length of hospital stay (r = −0.641, p = 0.014).

Conclusions: CogEvo demonstrated universal feasibility, convergent validity with MMSE and TMT, and clinically meaningful associations with length of hospital stay in acute stroke patients. Domain-level analyses suggest that planning ability may be particularly relevant to the discharge trajectory. CogEvo may serve as a feasible ICT-based complement to conventional cognitive assessment in the acute stroke setting. Prospective multicenter studies are warranted.

## Introduction

Stroke is a leading cause of disability worldwide, and post-stroke cognitive impairment (PSCI) is among its most prevalent and clinically significant sequelae. The incidence of PSCI ranges from 20% to 80%, with up to 90% of patients demonstrating some degree of cognitive disruption in the acute phase [1]. The 2023 American Heart Association scientific statement on PSCI explicitly recommends routine cognitive screening in the acute stroke setting, recognizing cognition as a core rehabilitation target from the outset of hospitalization [1,2].

Despite this recommendation, the implementation of systematic cognitive assessment in acute stroke units faces substantial practical barriers. The Mini-Mental State Examination (MMSE), the most widely used global cognitive screen, has well-documented limitations: it is susceptible to ceiling effects in patients with mild impairment, sensitive to educational attainment, and provides limited coverage of executive function and attention [3,4]. The Trail Making Test (TMT), which assesses processing speed (TMT-A) and cognitive flexibility/divided attention (TMT-B), complements the MMSE but requires sustained paper-based effort that may be beyond the capacity of medically unstable or neurologically impaired acute-phase patients [5,6]. In our experience, a substantial proportion of acute stroke patients are unable to complete the TMT due to motor deficits, attentional impairment, and fatigue.

CogEvo (Total Brain Care Co., Ltd., Japan) is a tablet-based computerized cognitive function assessment and training tool that evaluates five domains: orientation, attention, memory, planning, and spatial cognition. Originally developed for screening age-related cognitive decline and mild cognitive impairment in community-dwelling older adults, its reliability, validity, and two-factor structure have been established in that population [7-9]. Key practical advantages include a gamified touchscreen interface that reduces administration burden, independence from literacy, and potential for repeated assessments without practice effects [7]. However, whether CogEvo can be feasibly administered to acute stroke patients and demonstrates convergent validity in this setting has not been investigated.

This prospective pilot study therefore aimed to: (1) examine the feasibility of CogEvo administration in consecutive acute stroke patients; (2) assess correlations between CogEvo composite and domain scores and established cognitive measures (MMSE, TMT) as well as clinical variables (NIHSS, length of hospital stay, days from admission to assessment); and (3) characterize the profile of patients for whom CogEvo is applicable in the acute setting [10].

## Materials and methods

Study design and setting

This was a prospective, single-center, observational pilot study conducted at a stroke-specialized hospital in Japan, supported by the Showa University Academic Research Grant (fiscal year 2021). The study was approved by the institutional review board (approval date: August 26, 2021; approval number: 21-042-B) and conducted in accordance with the Declaration of Helsinki. Written or verbal informed consent was obtained from all participants.

Participants

Consecutive patients admitted for acute stroke (ischemic stroke, intracerebral hemorrhage, or subarachnoid hemorrhage) between September 2021 and August 2022 were screened. Inclusion criteria: (1) acute stroke confirmed by neuroimaging; (2) age 20-80 years; (3) ability to communicate to provide informed consent. Exclusion criteria: (1) visual or hearing impairment precluding assessment; (2) severe dementia preventing comprehension; (3) severe higher brain dysfunction precluding task execution (including severe hemispatial neglect, apraxia, or agnosia confirmed by clinical evaluation); and (4) sensory aphasia.

Assessments

CogEvo

CogEvo assesses five cognitive domains via touchscreen tablet: orientation (Reality Orientation), attention (Visual Search), memory (Flashlight), planning (Route 99), and spatial cognition (Just Fit). Domain scores are summed to yield a composite total score; higher scores indicate better cognitive function [7]. Assessments were conducted at a mean of 13.9 ± 7.5 days post-admission (range: 6-35 days).

MMSE

The 30-point Mini-Mental State Examination was used as a global cognitive screen. Scores ≤23 indicate dementia; scores 24-27 suggest mild cognitive impairment [10].

TMT-A and TMT-B

TMT-A and TMT-B assess processing speed and cognitive flexibility, respectively. Completion times in seconds were recorded. Patients unable to complete either part due to neurological impairment (motor deficit, severe attentional impairment, or comprehension difficulty) were excluded from TMT analyses. No imputation was performed.

Clinical data included age, sex, height, body weight, stroke type, lesion laterality, comorbidities, NIHSS at admission and discharge, days from admission to CogEvo assessment, length of hospital stay, and discharge destination.

Statistical analysis

Descriptive statistics were computed for all variables. Primary analyses: Spearman rank-order correlations between CogEvo composite score and each variable (N = 17 for MMSE, NIHSS, length of hospital stay, age, and days to assessment; complete case analysis for TMT). Exploratory secondary analyses: Spearman correlations between each CogEvo domain score and MMSE and length of hospital stay (N = 14, patients with complete domain data). As a post hoc sensitivity analysis suggested by an adviser, exploratory partial correlations between the CogEvo composite score and MMSE, TMT-A, and TMT-B were computed, adjusting for age and discharge NIHSS, using the same N = 14 subsample with complete domain-level data, to examine whether these associations were independent of demographic and stroke-severity-related factors. No correction for multiple comparisons was applied, given the exploratory nature; all results are hypothesis-generating. Analyses were conducted using JMP Pro version 16 (SAS Institute, Cary, NC). Although all 17 patients completed CogEvo, individual domain-level scores were fully extractable for 14 patients; the remaining three patients had incomplete domain records due to partial session data. Domain-level analyses, therefore, used N = 14.

## Results

Participant characteristics

Seventeen patients met eligibility criteria and completed CogEvo and MMSE assessments. Table 1 presents baseline characteristics. The mean age was 66.3 ± 14.9 years; 12 (70.6%) were male. Ischemic stroke was the most common diagnosis (64.7%). The median admission NIHSS was 3 (range 0-12). CogEvo was administered at a mean of 13.9 ± 7.5 days post-admission. Twelve patients (70.6%) were discharged home; the mean hospital stay was 19.2 ± 14.9 days.

**Table 1:** Baseline demographics and clinical characteristics (N = 17) NIHSS: National Institutes of Health Stroke Scale; SD: standard deviation.

Characteristic		Total (N = 17)
Age (years), mean ± SD	—	66.3 ± 14.9
Sex	Male	12 (70.6%)
—	Female	5 (29.4%)
Height (cm), mean ± SD	—	162.6 ± 9.9
Body weight (kg), mean ± SD	—	58.9 ± 12.3
Discharge destination	Home	12 (70.6%)
—	Transfer to facility	5 (29.4%)
Stroke type	Ischemic stroke	11 (64.7%)
—	Intracerebral hemorrhage	4 (23.5%)
—	Subarachnoid hemorrhage	2 (11.8%)
Lesion side	Left	8 (47.1%)
—	Right	8 (47.1%)
—	Bilateral	1 (5.9%)
Comorbidities	Hypertension	13 (76.5%)
—	Dyslipidemia	6 (35.3%)
—	Diabetes mellitus	3 (17.6%)
—	Atrial fibrillation	1 (5.9%)
NIHSS at admission, median (range)	—	3 (0–12)
NIHSS at discharge, median (range)	—	0 (0–5)
Days from admission to CogEvo assessment, mean ± SD	—	13.9 ± 7.5 (range: 6–35)
Length of hospital stay (days), mean ± SD	—	19.2 ± 14.9

TMT feasibility

Of the 17 enrolled patients, 12 (70.6%) completed TMT-A and 10 (58.8%) completed TMT-B. Seven patients (41.2%) were unable to complete TMT-B due to neurological impairment, including motor impairment of the dominant hand, severe attentional deficit, or comprehension difficulty. All 17 patients successfully completed CogEvo, demonstrating its broader feasibility across the spectrum of stroke severity.

Cognitive assessment results

Table 2 presents descriptive statistics. The mean CogEvo composite score was 1132.7 ± 585.5 points (range: 77-2232). The mean MMSE was 24.2 ± 5.1. Among completers, mean TMT-A was 49.3 ± 19.2 seconds (N = 12, range: 22-83 s), and mean TMT-B was 123.2 ± 68.7 seconds (N = 10, range: 43-231 s).

**Table 2:** Descriptive statistics for cognitive outcome measures a TMT values based on patients who completed the test (TMT-A: N = 12; TMT-B: N = 10). No imputation was performed. CogEvo: Cognitive function balancer; MMSE: Mini-Mental State Examination; TMT-A/B: Trail Making Test Part A/B; SD: standard deviation.

Measure	Mean	SD	Max	Min
CogEvo total score (points)	1132.7	585.5	2232	77
Orientation	238.0	138.9	—	—
Attention	185.1	85.7	—	—
Memory	238.8	210.9	—	—
Planning	200.3	106.6	—	—
Spatial cognition	270.4	146.1	—	—
MMSE (points)	24.2	5.1	30	13
TMT-A (seconds) a	49.3	19.2	83	22
TMT-B (seconds) b	123.2	68.7	231	43

Primary correlation analysis

Table 3 presents Spearman correlation results. CogEvo composite score was significantly positively correlated with MMSE (r = 0.693, p = 0.002, N = 17) and significantly negatively correlated with length of hospital stay (r = −0.518, p = 0.033, N = 17). In complete case analyses, CogEvo was significantly correlated with TMT-A (r = −0.580, p = 0.048, N = 12) and TMT-B (r = −0.648, p = 0.043, N = 10). Days from admission to assessment were not significantly correlated with CogEvo composite score (r = −0.247, p = 0.395, N = 14), suggesting that score variability was not primarily attributable to assessment timing.

**Table 3:** Spearman rank-order correlations between CogEvo total score and clinical/cognitive variables * p < 0.05. a TMT-A: complete case analysis (N = 12). b TMT-B: complete case analysis (N = 10). MMSE: Mini-Mental State Examination; TMT-A/B: Trail Making Test Part A/B; NIHSS: National Institutes of Health Stroke Scale; n.s.: not significant; CI: confidence interval (bootstrapped, 1000 iterations).

Variable	N	r	p-value	95% CI	Interpretation
MMSE	17	0.693	0.002 *	0.28–0.90	Significant positive
Length of hospital stay	17	−0.518	0.033 *	−0.79–−0.06	Significant negative
TMT-A (seconds) a	12	−0.580	0.048 *	−0.87–−0.01	Significant negative
TMT-B (seconds) b	10	−0.648	0.043 *	−0.90–−0.02	Significant negative
Age (years)	17	−0.420	0.093	—	Trend (n.s.)
NIHSS at discharge	17	−0.480	0.051	—	Trend (n.s.)
Days to assessment	14	−0.247	0.395	—	Not significant
NIHSS at admission	17	−0.100	0.697	—	Not significant

Exploratory domain-level analysis

Table 4 presents exploratory correlations between each CogEvo domain and MMSE and length of hospital stay (N = 14, complete domain data). Attention (r = 0.633, p = 0.015), planning (r = 0.668, p = 0.009), and spatial cognition (r = 0.664, p = 0.010) were each significantly correlated with MMSE. Planning was also significantly correlated with length of hospital stay (r = −0.641, p = 0.014), suggesting that this domain may carry particular prognostic relevance. Orientation and memory did not reach significance for either outcome. Scatter plots for the four significant domain-level associations are displayed in Figures 2A-2D.

Sensitivity analysis

Partial correlations adjusting for age and discharge NIHSS. Given that age and discharge NIHSS showed trend-level associations with the CogEvo composite score (Table 3), exploratory partial correlations were computed using the N = 14 subsample with complete domain-level data, adjusting for both covariates simultaneously. After adjustment, the correlation between CogEvo and MMSE was attenuated from r = 0.604 (p = 0.022) to r = 0.353 (p = 0.216). Similarly, the unadjusted associations with TMT-A (r = −0.433, p = 0.122) and TMT-B (r = −0.448, p = 0.108) were attenuated to r = 0.138 (p = 0.637) and r = −0.142 (p = 0.628), respectively, after adjustment. These findings suggest that, within this small subsample, part of the unadjusted association between CogEvo and conventional cognitive measures may be attributable to shared variance with age and neurological severity rather than representing fully independent convergent validity; given the limited sample size, these sensitivity analyses should be interpreted cautiously and considered hypothesis-generating rather than confirmatory.

**Table 4:** Exploratory Spearman correlations between CogEvo domain scores and MMSE/length of hospital stay (N = 14) * p < 0.05. All analyses are exploratory; no correction for multiple comparisons is applied. MMSE: Mini-Mental State Examination; LOS: length of hospital stay; n.s.: not significant. N = 14 (patients with complete domain-level CogEvo data).

CogEvo Domain	N	vs MMSE r	p	vs LOS r	p	Interpretation
Attention	14	0.633	0.015 *	−0.120	0.683	MMSE sig.
Planning	14	0.668	0.009 *	−0.641	0.014 *	Both sig.
Spatial cognition	14	0.664	0.010 *	−0.469	0.091	MMSE sig.
Memory	14	0.424	0.131	−0.356	0.212	n.s.
Orientation	14	0.303	0.293	−0.504	0.066	n.s.

**Figure 1:**
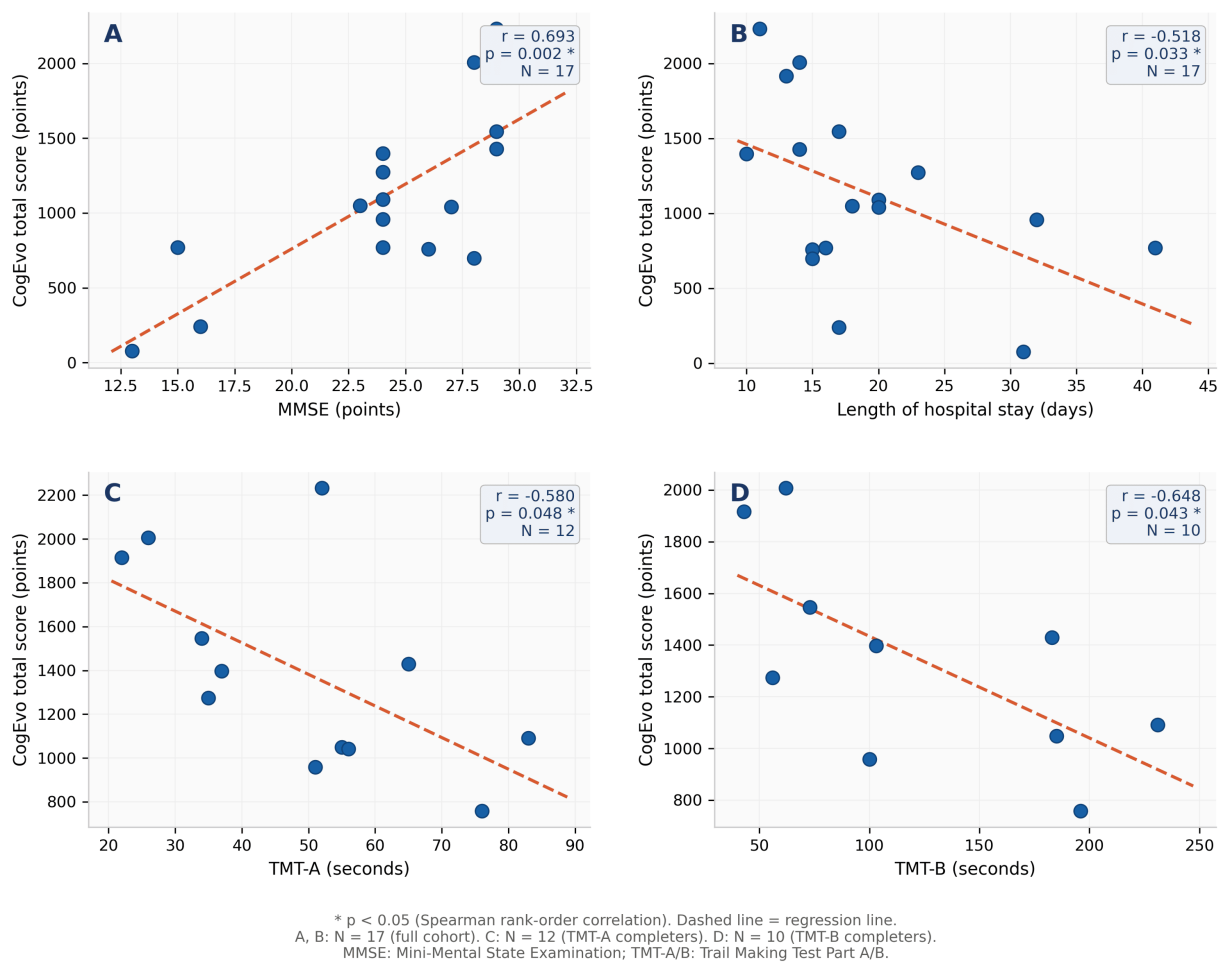
Scatter plots of CogEvo total score versus primary clinical and cognitive variables (A) CogEvo total score vs. MMSE (r = 0.693, p = 0.002, N = 17). (B) CogEvo total score vs. length of hospital stay (r = −0.518, p = 0.033, N = 17). (C) CogEvo total score vs. TMT-A (r = −0.580, p = 0.048, N = 12, complete case analysis). (D) CogEvo total score vs. TMT-B (r = −0.648, p = 0.043, N = 10, complete case analysis). Dashed lines indicate regression lines. MMSE: Mini-Mental State Examination; TMT-A/B: Trail Making Test Part A/B.

**Figure 2:**
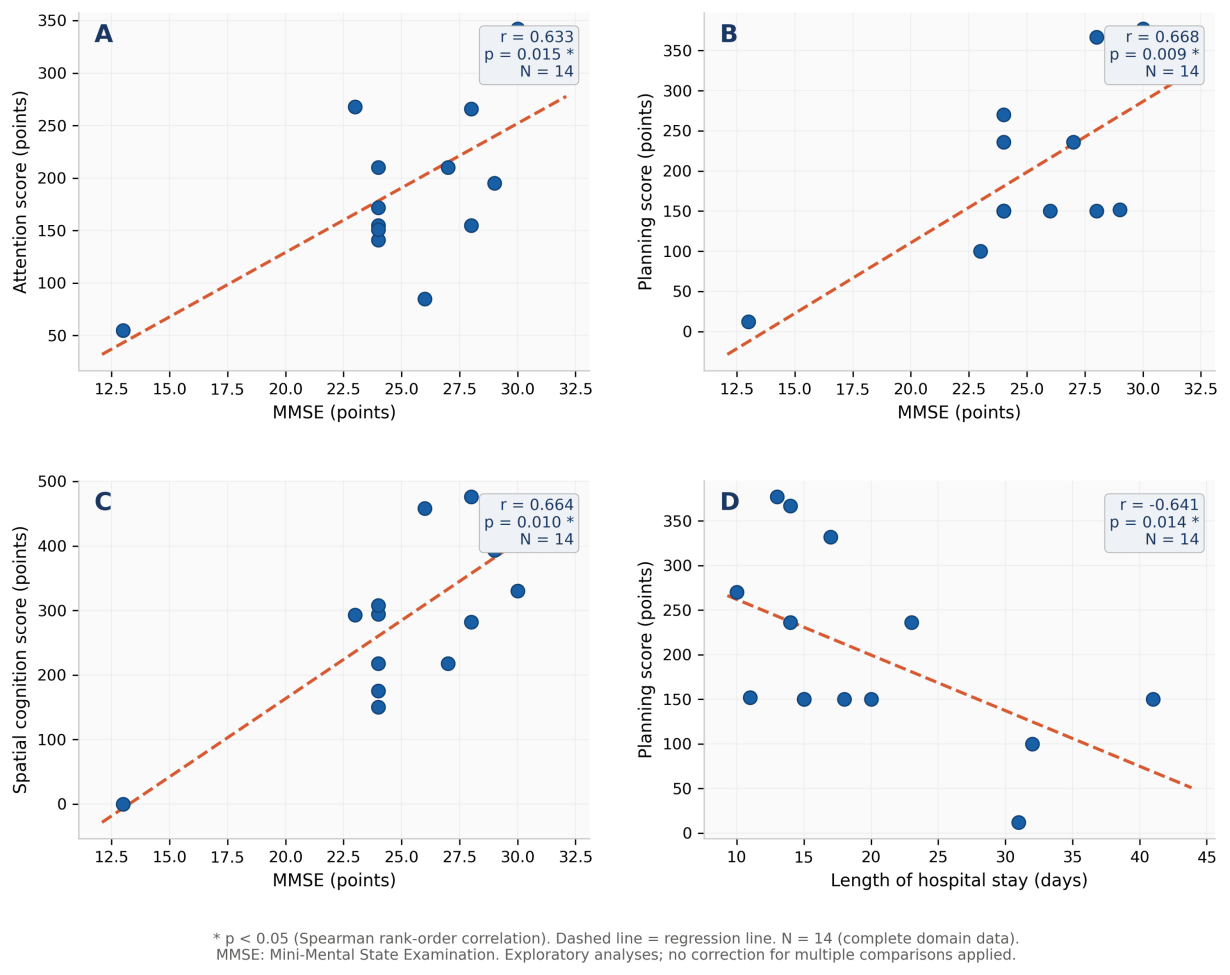
Scatter plots of CogEvo domain scores versus MMSE and length of hospital stay (exploratory analyses, N = 14) (A) Attention score vs. MMSE (r = 0.633, p = 0.015). (B) Planning score vs. MMSE (r = 0.668, p = 0.009). (C) Spatial cognition score vs. MMSE (r = 0.664, p = 0.010). (D) Planning score vs. length of hospital stay (r = −0.641, p = 0.014). Dashed lines indicate regression lines. All analyses are exploratory; no correction for multiple comparisons was applied. MMSE: Mini-Mental State Examination.

Figure 1 displays scatter plots for the primary correlation analyses: CogEvo total score vs. MMSE (Figure 1A), length of hospital stay (Figure 1B), TMT-A (Figure 1C), and TMT-B (Figure 1D). C represents TMT-A complete case analysis (N = 12); D represents TMT-B complete case analysis (N = 10). Figure 2 displays scatter plots for the four significant domain-level associations: attention vs. MMSE (Figure 2A), planning vs. MMSE (Figure 2B), spatial cognition vs. MMSE (Figure 2C), and planning vs. length of hospital stay (Figure 2D).

## Discussion

This prospective pilot study is the first to examine the applicability and convergent validity of CogEvo in acute stroke patients. Five principal findings emerge, each with implications for the clinical implementation of cognitive screening in acute stroke care [1]: (1) CogEvo was completed by all 17 patients, whereas only 10 (58.8%) completed TMT-B; (2) CogEvo correlated significantly with MMSE (r = 0.693, p = 0.002); (3) CogEvo was significantly associated with length of hospital stay (r = −0.518, p = 0.033); (4) among TMT completers, CogEvo correlated with TMT-A (r = −0.580, p = 0.048) and TMT-B (r = −0.648, p = 0.043); and (5) planning ability showed the most consistent domain-level associations with both MMSE and hospital stay.

The feasibility advantage of CogEvo over the TMT is clinically significant. Seven of 17 patients (41.2%) could not complete TMT-B, consistent with prior reports that paper-based neuropsychological tests are frequently infeasible in the acute phase due to motor deficits, attentional impairment, and fatigue [6]. CogEvo's touchscreen gamified format substantially reduces this barrier and enables cognitive screening in patients who are currently assessed as 'untestable' by conventional means [7]. A systematic review of computerized cognitive assessment tools in stroke patients confirmed that digital formats generally demonstrate acceptable feasibility and convergent validity with established paper-based measures, while also offering advantages in administration efficiency and reduced examiner burden [10]. Notably, CogEvo scores were not significantly correlated with admission NIHSS (r = −0.100), suggesting that CogEvo may detect cognitive impairment independent of neurological severity. This finding is consistent with recent evidence demonstrating that even patients with mild stroke (median NIHSS = 1) exhibit post-stroke cognitive impairment in up to 69% of cases within days of onset [16]. Patients who appear neurologically mild may still harbor deficits affecting motor learning efficiency during mobilization, transfer training, and gait practice, as cognitive status has been shown to significantly predict motor learning outcomes in stroke rehabilitation [13]. Furthermore, patients who have undergone repeated assessments may answer MMSE items from memory, and some patients experience resistance to formal paper-based testing; CogEvo's gamified format may provide a more authentic reflection of cognitive capacity in such cases [5].

The CogEvo-MMSE correlation (r = 0.693) confirms convergent validity, extending Ichii et al.'s foundational work to an acute stroke population [7]. The moderate rather than perfect correlation indicates that the two instruments do not measure identical constructs, consistent with prior observations that different cognitive screening tools capture distinct cognitive domains with varying sensitivity [4,5]. CogEvo is not subject to the ceiling effects or educational bias that limit the MMSE [9] and may detect impairment that the MMSE misses, particularly in executive and visuospatial domains where the MMSE is weakest [4]. It should be noted, however, that our exploratory sensitivity analysis adjusting for age and discharge NIHSS attenuated the CogEvo-MMSE correlation from r = 0.604 to r = 0.353 in the N = 14 subsample, suggesting that age and stroke severity may partially confound this association; this finding warrants replication in a larger sample before strong conclusions about CogEvo's independent convergent validity can be drawn.

The association between CogEvo and length of hospital stay (r = −0.518) is clinically meaningful. Domain analyses identified planning ability as independently associated with hospital stay (r = −0.641), suggesting that executive planning may influence rehabilitation participation and the duration of hospitalization. This interpretation is consistent with previous evidence demonstrating that cognitive impairments, particularly deficits in executive function, can negatively affect motor learning and rehabilitation outcomes after stroke [13]. Early identification of planning deficits may, therefore, help therapists anticipate barriers to rehabilitation participation and functional recovery, enabling timely intervention and individualized treatment planning. When attention or executive function deficits are identified, occupational therapists can adapt home exercise programs by simplifying procedural steps, incorporating external cues such as written instruction sheets or timer-based prompts, and clearly defining supervision requirements. CogEvo's visual and interactive output may also facilitate communication of cognitive findings to patients, family members, nurses, care managers, and community care providers, supporting continuity of care from acute hospital settings to home-based and community services [12].

This study has several notable strengths. To our knowledge, it is the first to prospectively examine CogEvo’s feasibility and convergent validity specifically in an acute stroke population, addressing a clinically important gap given the well-documented limitations of conventional paper-based tools in this setting. The use of consecutive, unselected patients minimized selection bias, and clearly defined inclusion and exclusion criteria support the transparency of the eligibility process. Concurrent administration of CogEvo alongside two independently validated reference measures (MMSE and TMT) allowed direct comparison of feasibility and convergent validity within the same patients. Additionally, the inclusion of exploratory domain-level and sensitivity analyses, despite the small sample size, provides a more nuanced and transparent interpretation of the findings than composite-score analysis alone.

Several limitations must be acknowledged. The sample size (N = 17) is small and from a single center, limiting statistical power and generalizability. The predominantly mild-to-moderate severity cohort (median NIHSS = 3) restricts applicability to more severe presentations. The single-time-point cross-sectional design precludes longitudinal tracking of cognitive recovery. Assessment timing was not standardized (range: 6-35 days), though days-to-assessment did not significantly correlate with CogEvo scores. Time from symptom onset to hospital admission was not recorded in this dataset; given that pre-hospital delay may influence both acute neurological status and subsequent cognitive presentation, this represents an additional limitation that should be addressed in future prospective studies. TMT analyses are based on N = 10-12; prior work has demonstrated that speed and executive complexity represent dissociable components of TMT performance, suggesting that our combined TMT-A/B findings should be interpreted with caution [14]. Domain-level analyses, including the exploratory partial correlation sensitivity analysis adjusting for age and NIHSS, are limited to N = 14 due to incomplete domain records in three cases; with only two covariates and 14 observations, these adjusted analyses are likely underpowered and susceptible to overfitting and should be regarded as preliminary. CogEvo was not compared against a comprehensive neuropsychological battery, limiting sensitivity and specificity estimates. CogEvo requires touchscreen tablet operation, and scores may be influenced by patients' familiarity with digital devices, upper limb motor function, and finger dexterity, factors particularly relevant in stroke patients with hemiplegia or fine motor impairment. Finally, CogEvo was developed in Japanese; cross-cultural and cross-linguistic applicability requires separate validation.

This pilot supports a prospective multicenter study with serial CogEvo assessments, a comprehensive neuropsychological reference standard, and longitudinal outcomes including functional independence and community reintegration at three and six months post-stroke. Serial computerized cognitive assessment has been shown to effectively map the trajectory of acute mild-stroke cognitive recovery [15], and future studies should exploit CogEvo's repeated-measurement capability to characterize individual recovery trajectories. Receiver operating characteristic (ROC) analyses should determine optimal composite and domain-level cutoff scores for detecting PSCI, given the high prevalence of cognitive impairment even among neurologically mild presentations [16]. Lesion-symptom mapping analyses should examine whether domain profiles differ by lesion location and laterality.

## Conclusions

In this pilot study of 17 acute stroke patients, CogEvo demonstrated universal feasibility, convergent validity with MMSE and TMT, and significant associations with length of hospital stay. Domain-level analyses suggest that planning ability may be particularly relevant to the discharge trajectory. Critically, CogEvo was administrable in patients unable to complete the TMT, the most common clinical scenario in the acute phase, highlighting its practical advantage. These findings support CogEvo as a promising ICT-based cognitive assessment complement for the acute stroke setting, particularly suited to mild-to-moderate presentations as a first-level screen. For occupational therapists, integrating CogEvo into acute stroke evaluation may not only improve the completeness of cognitive screening but also inform individualized rehabilitation planning toward meaningful functional recovery. Prospective multicenter studies with adequate sample sizes are needed to confirm and extend these results.
